# Selective Bias Virtual Screening for Discovery of Promising Antimalarial Candidates targeting Plasmodium N-Myristoyltransferase

**DOI:** 10.21203/rs.3.rs-3963523/v1

**Published:** 2024-02-20

**Authors:** Carolina Andrade, Bruna Katiele de Paula Sousa, Sunniva Sigurdardóttir, Catarina Bourgard, Joyce Borba, Leandro Clementino, Luis Carlos Salazar-Alvarez, Sophia Groustra, Rachael Zigweid, Monique Khim, Bart Staker, Fabio Costa, Leif Eriksson, Per Sunnerhagen

**Affiliations:** Federal University of Goias; Universidade Federal de Goiás; University of Gothenburg

## Abstract

Malaria remains a significant public health challenge, with *Plasmodium vivax* being the species responsible for the most prevalent form of the disease. Given the limited therapeutic options available, the search for new antimalarials against *P. vivax* is urgent. This study aims to identify new inhibitors for *P. vivax N*-myristoyltransferase (PvNMT), an essential drug target against malaria. Through a validated virtual screening campaign, we prioritized 23 candidates for further testing. In the yeast NMT system, seven compounds exhibit a potential inhibitor phenotype. *In vitro* antimalarial phenotypic assays confirmed the activity of four candidates while demonstrating an absence of cytotoxicity. Enzymatic assays reveal LabMol-394 as the most promising inhibitor, displaying selectivity against the parasite and a strong correlation within the yeast system. Furthermore, molecular dynamics simulations shed some light into its binding mode. This study constitutes a substantial contribution to the exploration of a selective quinoline scaffold and provides valuable insights into the development of new antimalarial candidates.

## Introduction

Malaria is a disease caused by protozoa of the *Plasmodium* genus, specifically the species *P. falciparum and P. vivax*^1^ that cause the highest rates of mortality and morbidity, respectively. In 2022, global estimates surpassed 200 million cases, resulting in over 600 thousand deaths^2^. Vivax malaria, the most geographically widespread form of the disease, is predominantly found in tropical and subtropical regions. Cases caused by *P. vivax* account for approximately 72% of all malaria episodes occurring outside the African continent^3^. Notably, around 73% of reported cases in the Americas are concentrated in Venezuela, Brazil and Colombia^2,4^.

*P. vivax* is biologically distinct from *P. falciparum* primarily due to the formation of hypnozoites, a latent stage that persists in the liver, posing a challenge for treatment due to the relapse episodes^5–8^. Additionally, *P. vivax* is characterized by its preference for reticulocyte infection^9,10^ and the early production of sexual stages (gametocytes) observed in peripheral blood^11,12^. An additional concern is related to the growing resistance of *P. vivax* to chloroquine^13–15^. Despite being a frontline treatment for vivax malaria since 1946, current evidence shows the circulation of resistant strains in endemic areas across various continents, including the Americas^16^. Evaluating drug resistance in *P. vivax* is particularly challenging due to the difficulties in *in vitro* culturing of the parasite. Distinguishing between treatment resistance and late relapses identified in *in vivo* assessments adds complexity to the analysis^17^. Given these challenges, there is a pressing need to explore new therapeutic options for *P. vivax*.

**Figure 1:**
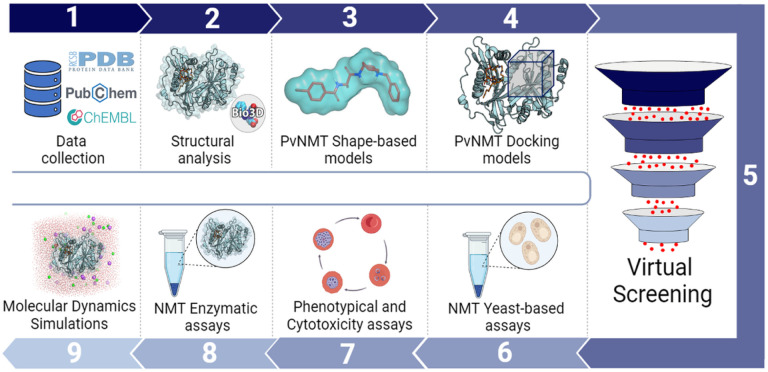
Overview of the study’s rationale for the development of new inhibitors targeting PvNMT. The process encompasses initial data acquisition from the Protein Data Bank (PDB) and literature-derived inhibitors, followed by structural analysis, shape-based and docking model development, and validation. Subsequently, a virtual screening campaign is conducted to prioritize compounds for *in vitro* validation, utilizing NMT yeast-based assays, malaria phenotypical and cytotoxicity assays, and NMT enzymatic assays. The most promising candidates undergo molecular dynamics simulations to analyse their binding modes.

The *N-* myristoyltransferase protein (NMT) plays a crucial role in catalyzing the transfer of the fatty acid myristate (C14) from the myristoyl-CoA molecule to the *N*-terminal glycine residue of numerous proteins^18^. In humans, this protein exhibits two isoforms, attracting attention in cancer research as important targets for cell survival^19^. In contrast, in various organisms, such as *Candida albicans*^20^, *Trypanossoma spp*.^21,22^, *Leishmania* donovani^23,24^, *Cryptosporidium parvum*^25^, and *Plasmodium spp*.^26–28^, which express a single isoform of the protein, NMT stands out as a promising drug target. This has prompted extensive research to discover new therapeutic agents for diseases associated with these organisms^29^. Studies revealed that many *Plasmodium* proteins need this post-translational modification during various life stages of the parasite^28^, which is important for drug development of inhibitors. While inhibitors for *Plasmodium* NMT are currently described, achieving parasite selectivity remains a challenge. Efforts are underway to enhance the selectivity of these inhibitors for more effective targeting of the parasite. Structural studies revealed that the *Plasmodium* NMT enzyme is capable of forming an alternate conformation in the peptide binding pocket which preferentially binds some inhibitors compared to the human enzymes thus providing a rational hypothesis for how to achieve selectivity^30^. Structural studies of these selective hits support the identification of a distinct conformation of the *Plasmodium* enzyme which binds compounds preferentially compared to the human NMTs^25,31^. In this study (Fig. 1), we employed structural analysis and virtual screening of millions of commercially available compounds using validated, robust, and predictive computational models as filters. This process enabled us to select 23 candidates for subsequent *in vitro* evaluation. Our approach involved experimental testing within a developed NMT yeast system, followed by *P. falciparum in vitro* phenotypic and cytotoxicity assays. Subsequently, the selected candidates underwent evaluation against NMT enzyme. To deepen our understanding, molecular dynamics simulations were conducted to elucidate the binding mode of the most promising candidates. Through this synergistic integration of computational models and experimental evaluation, we successfully identified LabMol-394 as a promising compound targeting the translational process of malaria parasites.

## Results

### *In silico* studies

#### Five high-affinity representative binding sites identified through structural analysis.

**Figure 2:**
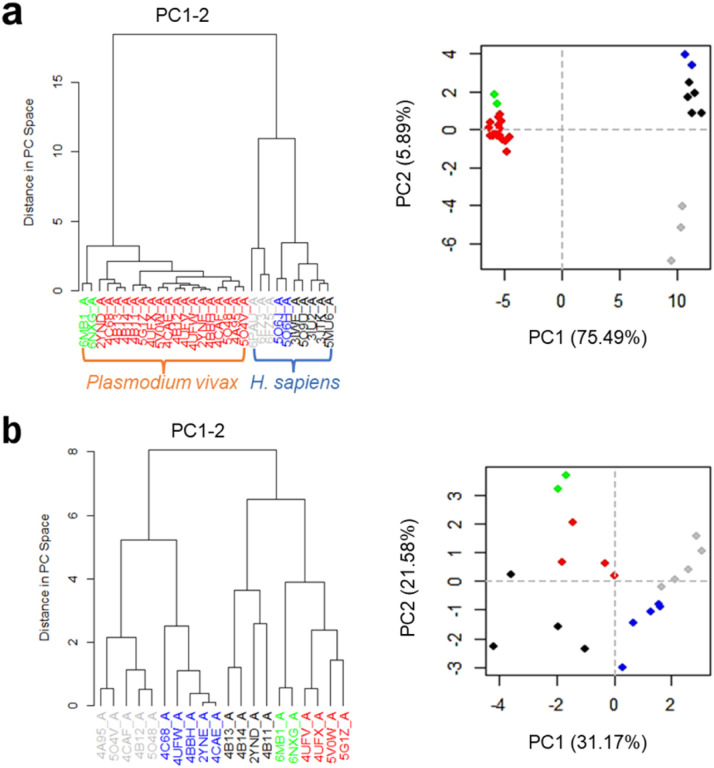
Principal Component Analysis (PCA) analysis depicting clustering of structural conformations of *Plasmodium* NMT. Panel a illustrates the comparison between PvNMT and HsNMT structures, revealing two distinct groups of conformations. Panel b showcases a heterogeneous separation among the PvNMT structures.

Utilizing data from PDB (https://www.rcsb.org/), we collected 59 structures of NMT from H *sapiens* (isoforms 1 and 2) and *P. vivax*. To conduct structural analysis using the Bio3D R package, we filtered the crystals based on the presence of a ligand, absence of mutations, and high resolution (≤ 2 Å), resulting in a total of 30 structures (10 for *H. sapiens* and 20 for *P. vivax)*. This comprehensive analysis involved aligning these structures followed by principal component analysis (PCA) to categorize similar conformations into clusters. Notably, our findings revealed distinct binding modes between *Plasmodium* compared to H *sapiens* (Fig. 2a). Building upon this insight, we focused on *Plasmodium* structures, identifying five clusters showcasing variations in conformation and ligand binding (Fig. 2b). Subsequently, we selected the most representative structures based on inhibition (Ki or IC_50_) from PDB IDs: 2YND, 2YNE, 4CAF, 4UFX and 6MB1. These structures served as the foundation for developing and validating both shape-based models and docking protocols.

### Validation of PvNMT Shape-based models

**Figure 3:**
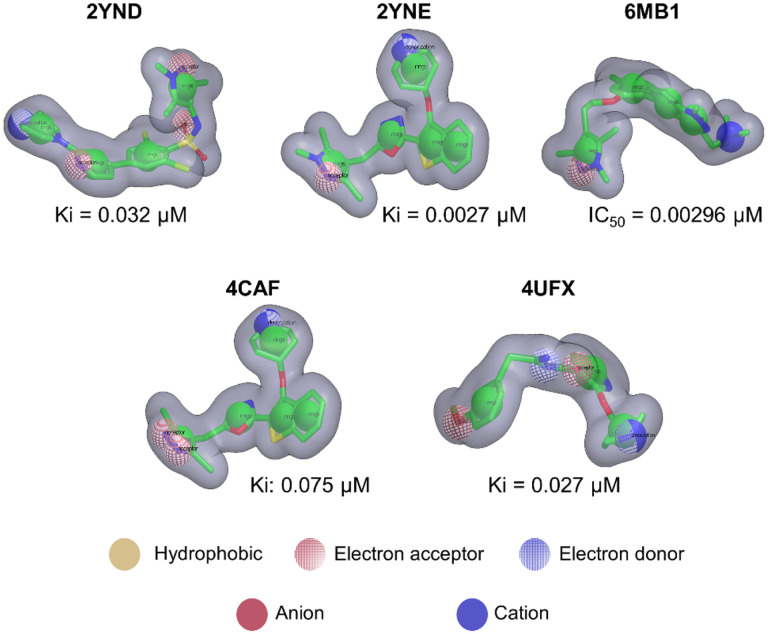
PvNMT shape-based models. Chemical structures, corresponding PDB-IDs and activities against *Plasmodium*NMT, utilized for the development and validation of the PvNMT shape-based models.

The selected representative structures were employed to construct and validate shape-based models, as depicted in Fig. 3. Notably, the application of RefTverskyCombo score yielded area under the curve (AUC) values ranging from 0.69 to 0.86 across all models. Particularly, the model generated using query PDB ID 2YNE demonstrated the highest performance, achieving an AUC of 0.86, along with an Enrichment Factor (EF) of 7.76 and BEDROC (Boltzmann-Enhanced Discrimination of ROC) score of 0.75 at the top 10%. These findings, summarized in Table 1, provide compelling evidence for the efficacy of the best-performing shape-based model, which exhibits satisfactory metrics and hence serves as a reliable filter in the virtual screening process.

### Selective bias docking and post -processing with MMGBSA correlates with PvNMT activity

To ensure the reliability of the docking program, validation was conducted using a prepared dataset of inhibitors and decoys along with the crystal structure of PvNMT PDB ID 2YNE, derived from the best-performing shape-based model. The results obtained from the top 10% of the ranked list yielded metrics including an AUC of 0.863, EF of 6.62 and a BEDROC of 0.65, all of which were considered satisfactory, validating the utilization of the Glide program for docking purposes. Subsequent redocking procedure involving the ligand at PDB ID 2YNE (2-(3-piperidin-4-yloxy-1-benzothiophen-2-yl)-5-[(1,3,5-trimethylpyrazol-4-yl) methyl]-1,3,4-oxadiazole), and its interaction with *Plasmodium* and human isoforms (I and II), revealed several key binding interactions. In *P. vivax,* the inhibitor primarily engages in π-stacking interactions with Phe105 and Tyr211, while also forming a hydrogen bond mediated by a water molecule with Ser319, and establishing a donor-salt bridge interaction between the piperidine ring and Leu410. This interaction yielded a docking score of −12.66 Kcal/mol and a predicted MMGBSA-ΔG of −99.37 Kcal/mol. Similarly, when interacting with HsNMT-1, the ligand engages in π-stacking interactions with tyrosines 296 and 420, forms a hydrogen bond with Ser405 (equivalent to Ser319 in *P. vivax),* and establishes a salt bridge interaction with Gln496 (analogous to Leu410 in *P. vivax)*. This interaction resulted in a docking score of −10.81 Kcal/mol and a significant predicted MMGBSA-ΔG difference of −28.60 Kcal/mol. Conversely, when interacting with HsNMT-2, the docking results suggested predominantly hydrophobic interactions at the peptide binding site, resulting in a docking score of −3.63 Kcal/mol and an MMGBSA-ΔG of −27.42 Kcal/mol. These findings are consistent with the IC_50_ for this ligand against human isoform 1 (60 ± 3 nM), demonstrating potential selectivity against *Plasmodium* (see Supplementary file, Fig. S1).

Furthermore, we evaluated the correlation between pK_i_ values and the predicted docking scores and MMGBSA values by calculating the correlation coefficient. Visual inspection of the data (see Supplementary file, Fig. S2) reveals that the most potent compounds exhibit docking score and MMGBSA values lower than - 11 Kcal/mol and - 80 Kcal/mol, respectively. Therefore, based on the docking metrics, redocking results and MMGBSA post -processing values obtained, we concluded that the program effectively predicts values closely aligned with the activity of the most potent compounds in the PvNMT dataset.

### Virtual Screening prioritizes 23 potential hits

**Figure 4:**
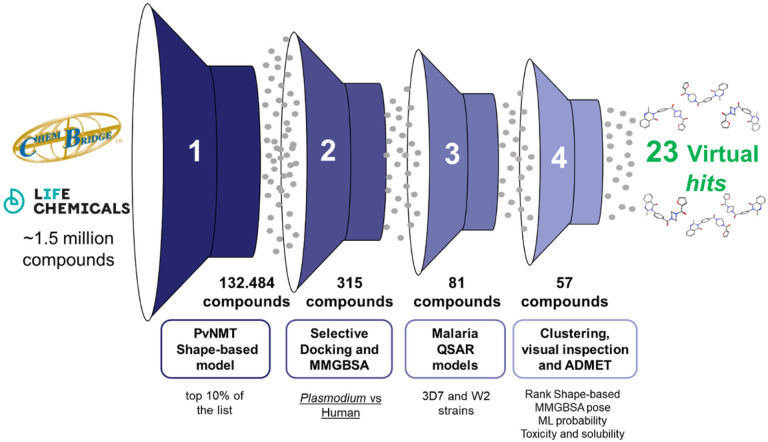
Molecular filters applied during virtual screening against PvNMT. The process the validated PvNMT shape-based model was employed as first filter, followed by the implementation of a selective docking protocol and the utilization of malaria QSAR models. Finally, clustering, visual inspections and assessment of ADMET properties were conducted.

With the models duly validated, the virtual screening was conducted applying the following filters (Fig. 4):

1. Utilization of the best molecular shape-based model for PvNMT, where the top 10% (131,982 compounds) of the list progressed to filter 2 for subsequent docking and selective rescoring between *P. vivax* and human isoforms.
2. Examination of the top 10% of the list based on the MMGBSA-ΔG score (≤ −70 kcal/mol). Compounds demonstrating a difference of ≤ −50 kcal/mol between HsNMT isoforms were singled out, resulting in the selection of 290 compounds.
3. Application of *P. falciparum* 3D7 (mandatory) and W2 classificatory QSAR models to predict the activity of compounds selected in the previous step (80 compounds), given the importance of the target in asexual blood stages.
4. Implementation of cluster analysis to identify representative chemotypes. Additionally, analysis of MMGBSA poses and evaluation of predicted ADMET characteristics were conducted. This comprehensive approach led to the final selection of 23 compounds (see Supplementary file, Table 1).

### Experimental evaluation

#### NMT yeast-based system for identification of potential inhibitors

**Figure 5:**
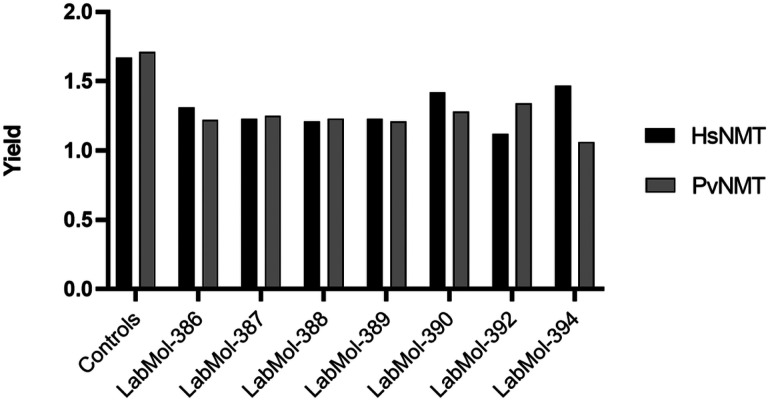
Growth ratio of compounds exhibiting an inhibitor phenotype against NMT yeast-based platform. Seven compounds demonstrated the inhibitor phenotype compared to the growth control.

We used a modified yeast strains system expressing the NMT targets of both H *sapiens* and *P. vivax* that allows a fast and cost-effective identification of potential selective inhibitors and cytotoxic compounds. Cells were cultivated and subjected to tests at 100 μM in triplicate for each strain (yPvNMT, yHsNMT1 and ScNMT). Optical density (OD) was measured over a 72-hour period. In this assay, compound inhibition is determined by the reduction in growth rate compared to the control (180 μL of cells and 20 μL of YPD medium) in the presence of compound. As a result, seven compounds (Fig. 5) presented an inhibition phenotype, with some demonstrating a degree of selectivity against the strain expressing *P. vivax* NMT strain, as evidenced by a reduction in growth ratio (yield) compared to the human strain. Notably, LabMol-394 exhibited a vivax phenotype, indicating potential selectivity towards the parasite isoform.

#### Four compounds exhibited antimalarial activity

To evaluate the antimalarial activity of the compounds, we conducted tests against chloroquine-sensitive P*f*3D7 and drug-resistant P*f*Dd2 and P*f*SB1A6 strains, using a concentration of 5 μM, as detailed in Table 2. Initially, we determined the percentage of parasite growth inhibition and subsequently generated dose-response (EC_50_) curves. Four compounds, LabMol-392, LabMol-393, LabMol-394, and LabMol-395, exhibited inhibition exceeding 80% against 3D7 strain parasites (see Supplementary file, Fig. S3 and S4), with EC_50_ values ranging from 0.098 to 1.10 μM. Notably, all compounds tested exhibited low cytotoxicity, as evidenced by the selective index (the ratio of selectivity to the parasite over mammalian cells) higher than 10 (except for LabMol 393), aligning with guidelines for malaria drug development^32^. Furthermore, hemolysis tests were conducted at a maximum concentration of 20 μM indicated no hemolytic activity for any of the compounds tested (Supplementary file, Fig. S5).

The most promising compound identified in the phenotypic screening is LabMol 395, demonstrating potent antimalarial activity below 0.17 μM against all three *P. falciparum* strains tested herein. Moreover, it exhibits remarkable selectivity for the parasite over mammalian cells (SI > 10), indicating significant potential for advancement in pre-clinical studies.

#### NMT enzymatic assays revealed potential inhibitors

**Figure 6:**
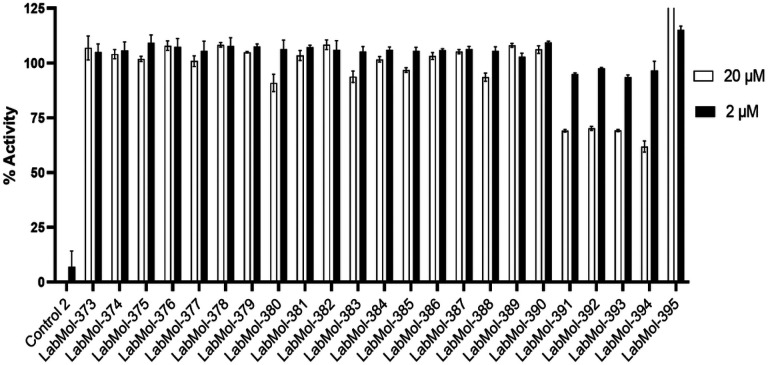
Two-concentration activity assay of 23 potential hit compounds. Control 02 is the known NMT inhibitor DDD85646.

We conducted a two-point NMT inhibition assay of the top 23 potential inhibitors. Activity percentages were determined at 2 μM and 20 μM to identify a potential activity of compounds. Unfortunately, IC_50_ calculations were not feasible due to compound solubility limitations. Nevertheless, the two-point enzymatic screen identified four promising compounds - LabMol-391, LabMol-392, LabMol-393 and LabMol-394 - which exhibited a minimum of 52% decrease in enzyme activity at a concentration of 20 μM against PvNMT (See Fig. 6). Regrettably, dose-response curves for the identified hits were hindered by compound precipitation at concentrations exceeding 40 μM. Subsequently, all compounds showing decreased activity were also tested against the human enzyme (HsNMT) in dose response studies ranging from 40 to 0.160 μM. Importantly, none of the compounds demonstrated inhibition of the human orthologue (HsNMT >40 μM, see Supplementary file, figures S6 and S7). These findings highlight four hit compounds that selectively inhibit PvNMT over HsNMT, thereby validating the efficacy of our computational selectivity approach.

#### Molecular dynamics simulations to analyze the binding mode of LabMol-394

**Figure 7:**
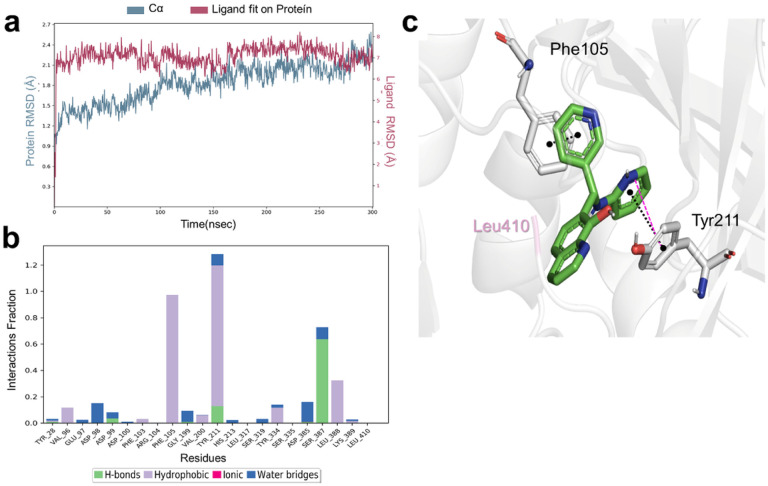
Dynamic interactions between PvNMT and LabMol-394 during a 300 ns MD simulation. **(A)** The root mean square deviation (RMSD) of the α carbon atoms of the protein in wine, and blue represents the ligand’s RMSD values. (B) Protein-ligand contacts, presenting the normalized fraction of interactions sustained during the simulation. (C) A representative MD pose is shown, highlighting interactions persisting for at least 50% of the simulation duration. LabMol-394 is shown in green, while interacting residues of PvNMT are highlighted residues. π-π stacking interactions are represented by black dashed dots, and π-cation interactions are represented by pink dashed lines. The key residue Leu410 is highlighted in light pink.

Molecular dynamics simulations of 300 nanoseconds were conducted to evaluate the stability and binding dynamics of LabMol-394 with PvNMT. During this simulation, several significant interactions were observed. Although LabMol-394 (Fig. 7) does not directly engage with a critical residue, Leu410, it consistently maintains π-stacking interactions with residues Tyr211 and Phe105, occurring approximately 51% and 63% of the time, respectively. Intriguingly, a persistent hydrogen bond with Ser387 and the hydroxyl group was observed for approximately 34% of the simulation duration, while a π-cation interaction with Tyr211 and nitrogen of the pyridine ring was maintained for approximately 51% of the time.

## Discussion

The process of discovering compounds with biological activity is a significant challenge, demanding the application of robust and predictive methodologies to ensure reliable outcomes. Bioinformatics and cheminformatics analyses serve as foundational tools in the search for novel antimalarial agents. Their effectiveness is evident through validation and research efforts led by esteemed organizations such as MalDA, MMV, the Bill and Melinda Gates Foundation, and the pharmaceutical industry^33^.

A crucial aspect of antimalarial drug discovery involves the identification of novel targets essential to the parasite’s life cycle and conserved among various *Plasmodium* species. This strategy aims to encompass species previously overlooked as harmful to humans^34^. Consequently, the integration of computational strategies plays a crucial role in the discovery process, enabling the exploration of innovative mechanisms of action for potential new antimalarials.

The NMT enzyme was validated as a target for antimalarial drugs in 2014^30^ with many inhibitors initially repurposed from other organisms^21,35–37^ and through High Throughput Screening (HTS) campaigns^38,39^. The latest NMT inhibitors, highlighted by Rodríguez-Hernández and colleagues, particularly compounds 12b and 30a from the series of hybrids of DDD85646^30^ and IMP-1002^40^ PvNMT inhibitors studies, demonstrated significant potency *in vitro* against PvNMT, with IC_50_ of 0.0368 μM and 0.089 μM, respectively. However, their efficacy against hypnozoites and schizonts was observed to be in the low micromolar range^31^.

In our study, we initially conducted an analysis of structural conformations within a collection of crystal structures containing the new PvNMT inhibitor. This analysis allowed us to discern the various binding modes employed by the inhibitors. In parallel, a gathered set of compounds from the literature that had been tested against PvNMT, rigorously validating our shape-based and docking computational models, in accordance with established good practices^41,42^. Subsequently, a virtual screening campaign was conducted. Our approach involved utilizing a predicted ΔG difference between *Plasmodium* and human proteins as a filter to reevaluate the docking scores. This strategy enabled us to prioritize compounds that showed potential selectivity for the parasite. As a result, we identified and prioritized 23 compounds with promising characteristics for further investigation.

During the experimental validation phase, seven compounds exhibited a phenotypic effect against the modified yeast NMT strains. Next, the compounds were assessed for their efficacy against *P. falciparum* strains, considering its significant identity (> 80%) of NMT between the species^43^. Notably, in *P. falciparum* strains assays, four of these compounds demonstrated significant activity against three parasite strains, all at a single concentration of 5 μM, without any indications of cross-resistance and no signs of cytotoxicity (< 50 μM) or hemolysis, which are indicative of a favorable chemical safety profile. It’s important to consider that the observed micromolar activity against the parasites may be influenced by the compounds’ permeability to erythrocytic cells. Moreover, various efflux pumps, such as *mdr1* and *mrp1* in *Plasmodium,* known for their role in resistance mechanisms^44–46^, could affect the activity of these compounds. In yeast cells, the primary efflux pump for drugs, the gene *PDR5,* was deleted in the strains we used^47^. This deletion leads to an increase in the concentration of compounds inside the cells, potentially explaining the divergent results observed for some compounds. One standout compound from these assays, LabMol-395, displayed an antimalarial efficacy below 0.17 μM against *P. falciparum* strains examined. However, this particular compound did not exhibit a yeast phenotype against NMT, indicating that its antimalarial activity likely involves a different target.

On enzymatic NMT assay, LabMol-394 exhibited modest activity, resulting in a 52% decrease in the activity of PvNMT at a concentration of 20 μM, displaying selectivity towards vivax over the human isoform. Molecular dynamics simulations revealed that LabMol-394 demonstrated interactions comparable to PvNMT inhibitors, including those used in the shape-based modeling. However, it lacked interaction with Ser319, previously suggested as a crucial residue in the proposed mechanism of NMT inhibition^30^. Throughout our simulations, LabMol-394 did not engage with a crucial residue, Leu410. However, it sustained significant n-stacking interactions with Tyr211 and Phe105 residues, while also forming a hydrogen bond with Ser387. Although Ser387 is not currently associated with inhibition in the literature, we hypothesize it as a potential mechanism.

Chemically, LabMol-394 features a quinoline scaffold extensively documented for its diverse biological activities^48^. This scaffold is particularly renowned for its well-established antimalarial effects, primarily attributed to its inhibition of β-hematin and the formation of an irreversible complex with the heme group^49^. These actions disrupt the development of the parasite in both liver and red blood cells. Notably, recent reports unveiled a novel quinoline inhibitor targeting the translation elongation factor 2 (PfEF2), demonstrating multistage antimalarial properties and currently progressing through clinical stages^50–53^. This discovery underscores the significance of heterocycles as crucial sources of chemical activities and introduces new mechanisms of inhibition aimed at pivotal targets in the *Plasmodium* life cycle.

The prioritized candidates in this study were experimentally validated and the results demonstrated a correlation between antimalarial activity and the absence of cytotoxicity. LabMol-395 emerged as promising candidate for target identification, due to its favorable antimalarial profile. Furthermore, enzymatic assays revealed LabMol-394 as the most promising PvNMT inhibitor, exhibiting selectivity against the parasite. Remarkably, there was a good correlation between the results obtained from the yeast system and enzymatic assays in NMT. This study makes a significant contribution to the exploration of the quinazoline scaffold, showcasing activity against *Plasmodium vivax* NMT and offering prospects for further optimization and development.

## Methods

### Computational

#### Structural analysis

For structural analysis, we utilized the Bio3D R package^54,55^, focusing on crystals with assigned ligand, no mutations and showed high resolution (≤ 2 Å). This analysis comprised the alignment of these structures and then principal component analysis (PCA) of the conformations was performed.

#### Data collection and preparation

To distinguish active compounds from inactive ones, we constructed and validated shape-based models. Initially, compounds tested against the enzymes of both *P. falciparum* and *P. vivax* were collected from the literature^24,35,56–59^. Subsequently, employing the protocol outlined by Fourches and colleagues^60,61^, we processed an initial set of 152 compounds. Briefly, the hydrogens were explicitly included while excluding counter ions, inorganic salts, polymers, mixtures, and organometallic compounds. We set a threshold for inhibitor activity considering compounds with Ki or IC_50_ values ≤1 μM as active and those with values ≥ 1 μM, as inactives. Additionally, specific chemotypes, such as aromatic and nitro groups, were standardized and duplicates were analyzed following the criteria: (i) entries with identical reported outcomes were consolidated, keeping only one, while the redundant entry was eliminated, and (ii) if duplicates exhibited inconsistent biological activity, both entries were removed from the dataset. Subsequently, the final dataset includes 137 unique compounds, comprising 67 inhibitors and 70 non-inhibitors. To augment the chemical diversity of the dataset, we generated 36 decoys for each inhibitor. The dataset, including actives, inactives and decoys, underwent appropriated hydrogen protonation state on neutral pH (7.4) using the Open Babel program^62^. Subsequently, for each molecule, 200 conformers were generated using the OMEGA^63^ program, and AM1BCC charges^64,65^ were estimated using the QUACPAC software^66^. Validated shape-based models were then built using the ligand conformations extracted from the Protein Data Bank (PDB) and employed as queries at the ROCS v. 3.4.2.1 program^67^.

#### Shape-based model development and validation

To assess the predictive performance of the shape-based models, the following metrics were analyzed: Receiver Operating Characteristic (ROC) curve, which provides a graphical representation of the true positive rate (sensitivity) against the false positive rate (1-specificity); Area Under the ROC Curve (AUC), which quantifies the probability of an inhibitor being ranked higher than an inactive compound when compared to a random selection by the *query*^41,68^; Boltzmann-Enhanced Discrimination of ROC (BEDROC), which uses an exponential decay function to assign weights to actives that ranked higher in the list^69^; Enrichment Factor (EF), that evaluates the fraction of actives found at the Top *n*% of the ranking compared to a random selection^70,71^. The statistical metrics were calculated using the following equations:

(1) $$AUC=\sum i\left[\left(Se_{i+1}\right)\left(Sp_{i+1}-Sp_{i}\right)\right]$$

(2) $$\text{BEDROC}=RIE\frac{R_{\alpha }\text{sin}\left(\frac{\alpha }{2}\right)}{\text{cosh}\left(\frac{\alpha }{2}\right)-\text{cosh}\left(\frac{\alpha }{2-\alpha R_{\alpha }}\right)}+\frac{1}{1-e^{\alpha \left(1-R_{\alpha }\right)}}\approx \frac{RIE}{\alpha }+\frac{1}{1-e^{\alpha^{\prime }}}$$

$$if\alpha R_{\alpha }\ll 1\text{and}\alpha \neq 0$$

(3) $$EF^{x\text{\%}}=\frac{\setminus \text{ raisebox}1\text{ex \$}Hits^{x\text{\%}}\;\text{selected}\$/\setminus \text{ raisebox }-1ex\text{\$}N^{x\text{\%}}\;\text{selected}\$}{\setminus \text{raisebox}1\text{ex\$}Hits{\;}_{\text{total}}\text{\$}\wedge \text{raisebox}-1ex\text{\$}N_{\text{total}}\text{\$}}$$

#### Selective bias docking and post-processing with MMGBSA

The protein structures of *Plasmodium* (PDB ID: 2YNE)^30^ and H *sapiens* (PDB ID: 5O6J)^72^ were processed in the Protein Preparation Wizard module^73^ within Maestro (Schrödinger 2021–4, http://www.schrodinger.com). This protocol includes addition of hydrogen atoms, calculation of ionization states at physiological pH (7.4 ± 0.5) using the Epik program^74,75^, and removal of structural waters with a distance greater than 3 Å from the side chains. Subsequently, protein hydrogen bond assignments and protonation states were refined using PROPKA, followed by restrained minimization using the OPLS-2005 force field^76,77^. Ligand preparation was carried out in the LigPrep module in Maestro (Schrödinger 2021–4, http://www.schrodinger.com), with ionization states set at physiological pH (7.4 ± 0.5) using the Epik program. Conformers were generated, retaining the stereoisomerism, and the geometry was minimized using the OPLS-2005 force field. Three-dimensional coordinates of the grid were constructed using the Receptor Grid Generation (Schrödinger 2021–4, http://www.schrodinger.com) module at the region of the peptide binding site reported in the literature for *P. vivax*^30^ (grid coordinates x = 24,29, y = 43,20, z = 64,66) and *H. sapiens*^72^*(grid* coordinates x = −18,31, y = 4,55, z = −19,80), have a size of 10 A. Docking validation was performed with the Glide program^78^ (Schrödinger 2021–4, http://www.schrodinger.com) using the PvNMT dataset prepared and protein structure of PvNMT. Standard precision (SP) was utilized, employing a score function that uses an exhaustive sampling search, recommended for virtual screenings campaigns^79^. Statistical metrics including AUC, EF and BEDROC were computed using an *in-house* workflow in KNIME^80^ to assess the robustness of the docking program. The MMGBSA rescoring method was performed in the Prime module version 3.0 (Schrödinger 2021–4, http://www.schrodinger.com). This module computes the energy of the ligand-protein complex in solvent from the initial docking poses. Rescoring was applied during protocol validation and during virtual screening for the Top 10% of the ranked list. The system was composed of implicit solvent of the VSGB2 model^81^, parametrized on OPLS-2005 force field. The hierarchical sampling method was employed, with active site residues kept rigid in the first cycle and site conformations explored in the second cycle until finding the lowest ΔG value expressed in Kcal/mol^82,83^.

#### Virtual Screening

Following the validation of shape-based models and docking, augmented by MMGBSA rescoring, the virtual screenings were carried out using the Core library and Express-Pick collection stock libraries from the ChemBridge database ( https://www.chembridge.com/screening_libraries/) and Life Chemicals Antimalarial Screening Libraries (https://lifechemicals.com/screening-libraries/targeted-and-focused-screening-libraries/antimalarial-screening-libraries). These combined libraries encompass over 1.5 million compounds in total. These data were compiled and processed following the mentioned protocol for ligand preparation. The first filter was the best shape-based model for PvNMT, where the Top 10% of the list went to the selective docking and rescoring filter between *Plasmodium* and human NMT. The Top 10% analyzed with the MMGBSA-ΔG threshold ≤ −70 Kcal/mol for PvNMT and presents a difference of ≤ −50 Kcal/mol against HsNMT. The compounds selected in the previous step were predicted by the 3D7 and W2 malaria machine learning models(described in^84^), considering that the target is essential during the asexual blood stage. For the final selection process, cluster analysis was performed using DataWarrior software^85^ to identify representative chemotypes and avoid redundancy by excluding very similar compounds. Additionally, a medicinal chemistry visual inspection of the MMGBSA pose was performed, and predictions of ADMET (Absorption, Availability, Metabolism, Excretion and Toxicology) were made using the SWISS-ADME server ( http://www.swissadme.ch/)^86^. Finally, the prioritized candidates (See Supplementary Data file 1) were purchased and validated through *in vitro* assays using yeast-modified strains expressing *P vivax* and human NMT, as well as *P. falciparum* 3D7, Dd2 and SB1A6 strains, cytotoxicity and enzymatic assays to comprehensively evaluate the compounds’ efficacy and safety profiles.

#### Molecular Dynamics simulations

The simulations were performed in the Desmond program version 6.9^87^ on the Maestro platform (Schrodinger 2021–4, http://www.schrodinger.com). The system for the 300 nanoseconds simulation was built in the System Builder module and used the explicit solvent (H2O) model of the TIP3P type^88,89^, the periodic boundary conditions of the orthorhombic type with water buffer at 10 A around the protein. Ion’s sodium (Na+) and chlorine (Cl^−^) were added to neutralize the system, whose final concentration was NaCl at 150 mM. The system was minimized in a cubic box using the OPLS4 force field^90^ and the isothermal-isobaric NPT ensemble was used, in which the number of particles, pressure and temperature in the system were constant. To adjust the temperature, the Nose-Hoover thermostat^91^ at 300 K, and the Martyna-Tobias-Klein barostato^92^ at 1.01325 pressure was used. Data processing of Root Mean Square Deviations (RMSD), Root Mean Square Fluctuation (RMSF), and protein-ligand interactions were obtained in the Simulation Interactions Diagram module.

Trajectory cluster analysis was performed using the Trajectory clustering module on Maestro (Schrodinger 2021–4, http://www.schrodinger.com) to obtain the most representative conformations of the lowest energy of the most populated cluster.

### Experimental

#### Construction of plasmids and yeast -modified strains

By codon usage optimization for expression in *Saccharomyces cerevisiae,* the coding sequences of NMT from H *sapiens* and *P. vivax* (here referred to as HsNMT and PvNMT, respectively) were synthesized, amplified by PCR, and subsequently, cloned into the *Bam* HI-*Psf* I sites of the yeast expression vector pCM188-URA3 (EUROSCARF), resulting in the generation of the following constructs: pCM188-URA3-HsNMT and pCM188-URA3-PvNMT. For more details on these constructs, please review the publication from Bilsland, E. and colleagues^93^. The DNA constructs, verified by sequencing, were used in this work for the yeast-based functional complementation system. *S. cerevisiae* strains and plasmids used are listed in Table 3.

#### Phenotypic analysis using Bioscreen

YPD media (20% glucose,2% peptone, and 1% yeast extract) was used for routine culturing of strains. Frozen stocks on exponential growth phase were transferred to Falcon tubes with YPD media. Growth was maintained for an hour in a rotary shaker at 30°C at 200 rpm. Before the start of experiment, the medium was removed, and the pellet resuspended in new media to a final OD_600_ of 1.0. Subsequently the cultures were diluted to an OD_600_ of 0.1 into 100-well honeycomb plates (Labsystems Oy) pre-aliquoted with the appropriate media and tests. Strains were cultivated for 3 days with the low shaking setting at 30°C with 20 minutes measurement intervals using a wide band filter (450 to 600 nm) using Bioscreen C (Labsystems Oy). At the end of the experiment, the data were treated and normalized in the PRECOG program^94^. Then an R script was used using a growth curve library, to compute the cell yield (K) values, which was the parameter that best fits the data^95^. The data was subsequently exported to the GraphPad Prism 9 program, and the growth of tests and control growth were analyzed by the non-linear logistic regression model.

#### Phenotypic antimalarial assays

The antimalarial activity assay was assessed on *P. falciparum* 3D7 (chloroquine-sensitive), Dd2 (chloroquine-resistant) and SB1 (atovaquone-resistant) strains^14^. The parasites were cultivated in RPMI 1640 medium (SIGMA-ALDRICH), supplemented with hypoxanthine 0.005%, glucose 0.2%, sodium bicarbonate 0.2%, O^+^ red blood cells (RBCs) and A^+^ human plasma 10%. The cultures were incubated at 37°C in a low oxygen environment (3% O2, 5% CO2, and 92% N2) as described by Trager et al.^96^ Drug inhibition assays were performed as previously described^97^. Briefly, synchronizations with 5% D-Sorbitol solution were performed at 48-hour intervals before experiments to allow incubations with > 90% of the parasites in the ring stage. Assays were performed in a 96-well plate, with 0.5% parasitemia and 2% hematocrit, in the presence of 5 μM of compounds or the drug vehicle (DMSO), as a control. Artesunate was used as an antimalarial standard. After 72 hours of incubation, parasitemia was assessed by fluorometry using SybrGreen fluorescent dye^98^. The plates were read in a CLARIOStar plate reader (BGMtech) by fluorescence at 490 nm excitation and 540 nm emission wavelengths. The growth inhibition values were expressed as percentages relative to the drug-free control, and EC_50_ values were calculated by plotting Log dosing vs growth inhibition (expressed as percentages relative to the drug-free control) using GraphPad Prism 8. The experiments were carried out in three independent assays.

#### Hemolysis assay

The hemolysis assay was carried out according to Wang et al. (2010)^99^ with modifications. Suspensions of erythrocytes (2% hematocrit) were incubated with the compounds at 20 μM or the drug vehicle (DMSO), as a control, at 37°C, 5% CO_2_, for 4 hours. The reaction mixtures were centrifuged at 1000g for 5 minutes, and the absorbance of the supernatants was measured at 540 nm using a Biotek Synergy-HT spectrophotometer. The hemolytic rate was calculated in relation to the hemolysis of erythrocytes in 1% of Triton X100, which was taken as 100%, using GraphPad Prism 8. The experiments were carried out in triplicate, relative to two independent assays.

#### Cytotoxicity assays

The cytotoxicity was evaluated using the MTT (3-[4,5-dimethyl-thiazol-2-yl]-2,5-diphenyltetrazolium chloride) reduction assay for quantification of cellular reductase enzymes activity as an indirect measurement of cell viability using human hepatocarcinoma cell line (HepG-2) and fibroblast-like cell lines derived from monkey kidney tissue (COS-7 cells^100^). Briefly, the cells were cultured in Dulbecco’s Modified Eagle Medium supplemented with 10% heat-inactivated fetal bovine serum and 1% antibiotic-antimycotic solution (10,000 UI of penicillin, 10 mg of streptomycin in 0.9% sodium chloride; Sigma Chemical Co., Saint Louis, USA) at 37°C and 5% CO_2_. The assays were conducted in a 96-well plate at a density of 10^4^ cells per well. The cells were then incubated with a serial dilution of the drugs, starting at 100 μM, along with non-treated controls, for 72 hours. After the drug treatment, MTT was added to the wells, and absorbance readings were obtained using a CLARIOStar plate reader (BGMtech) at a wavelength of 570 nm (OD570). Cellular viability was expressed as a percentage relative to the vehicle-treated control. The CC_50_ was calculated by plotting a log dose vs. viability curve using GraphPad Prism 8. The experiments were performed in two independent assays.

#### Enzymatic assays

To measure the activity of the purified PvNMT, an activity assay was adapted from Goncalves et al, 2012^101^. A fresh stock of 4x assay buffer was made consisting of 9.2 mM potassium phosphate (KH_2_PO_4_), 69.7 mM sodium phosphate (Na_2_HPO_4_), 2 mM EDTA, and 0.04% TritonX-100 at pH 7.0. Fresh working stock solutions were made by adding DMSO for a final concentration of 1% or 5% and a concentration of 1x assay buffer. Another working stock solution was made by adding DMSO for a final concentration of 10%, without assay buffer. The test compound solutions were made from the 20 mM stock. The test compounds were diluted in 10% DMSO to make a 10mM stock. From the 10 mM stock, the test compounds were further diluted into 20 μM and 2 μM stocks in 10% DMSO. A 10 μM stock of a control inhibitor, known as Control 02(DDD85646), was made, and then further diluted in 10% DMSO to make a 2 μM solution. 10 μL of the test compounds were plated in triplicates in a black, closed bottom 96-well plate (Greiner Bio-One). For the negative and positive control, 10 μL of 10% DMSO was plated in quadruplicates. 10 μL of the control inhibitor, Control 02, was plated in quadruplicates. The PvNMT protein was diluted in 1% DMSO containing 1x assay buffer for a final concentration of 10 μM. The PvNMT protein was diluted further in 1% DMSO, and 50 μL was added to each well in the 96-well plate for a final PvNMT concentration of 25 nM. For the negative control, 50 μL of 1% DMSO containing 1x assay buffer was added to the well instead of the PvNMT protein. The reaction was initiated by adding 50 μL of reaction substrate containing 10 μM PfARF (Gly-Leu-Tyr-Val-Ser-Arg-Leu-Phe-Asn-Arg-Leu-Phe-Gln-Lys-Lys-NH2 purchased from Innopep in San Diego, California), 10 μM Myr-CoA (myristoyl coenzyme A purchased from purchased from Med Chem 101 LLC in Plymouth Meeting, Pennsylvania), and 8 μM CPM (7-Diethylamino-3-(4’-Maleimidylphenyl)-4-Methylcoumarin purchased from Thermo Scientific Life Technologies in Grand Island, New York) to each well. Fluorescent readings were immediately taken of the plate using Spectra M2 plate reader (Molecular Devices) with excitation at 385 nm and emission at 485 nm. Reading continuously were taken in one-minute intervals for 45 minutes. Background fluorescence and noise were determined by replacing each constituent of the reaction individually with 1% DMSO containing 1x assay buffer, and values were deducted from experimental samples. The percent inhibition of each test compound was calculated using Prism (GraphPad Software, Inc).

## Figures and Tables

**Table 1:** Validation of shape-based models employing different queries and *RefTverskyCombo score*.

Queries	AUC	TOP 1%		TOP 5%		TOP 10%	
		EF	BEDROC	EF	BEDROC	EF	BEDROC
2YND	0.77	5.66	0.21	4.48	0.23	3.28	0.29
**2YNE** *	**0.86**	**37.00**	**0.88**	**17.63**	**0.74**	**7.76**	**0.75**
4CAF	0.73	1.49	0.08	3.58	0.13	3.13	0.21
4UFX	0.84	23.88	0.65	14.93	0.68	7.61	0.72
6MB1	0.69	7.46	0.20	3.88	0.19	2.09	0.22

AUC: Area under the ROC curve; EF: Enrichment Factor; BEDROC: Boltzmann-Enhanced Discrimination of ROC.

* Selected model.

**Table 3:** Plasmids and strains used in this study.

Plasmid	Features	Derived from	Source
pCM188-URA3	CEN; URA3; TetO2 promoter	-	EUROSCARF
pCM188-URA3-HsNMT	*Homo sapiens* NMT	pCM188-URA3	Ref^74^
pCM188-URA3-PvNMT	*Plasmodium vivax* NMT	pCM188-URA3	Ref^74^
BY4741	*MATa his3Δ1 leu2Δ0 met15Δ0 LYS2 ura3Δ0*		EUROSCARF
*nmt1Δ/NMT1*	*nmt1::KanMX/NMT1 MATa/MATα his3Δ1/his3Δ1 leu2Δ0/leu2Δ0 met15Δ0/MET15 LYS2/lys2Δ0 ura3Δ0/ura3Δ0;* Kan/G418		EUROSCARF
*nmt1Δ/NMT1_p*	*pdr5::HISMX/PDR5 nmt1::KanMX/NMT1 MATa/MATα his3Δ1/his3Δ1 leu2Δ0/leu2Δ0 met15Δ 0/MET15 LYS2/lys2Δ0 ura3Δ0/ura3Δ0;*Kan/G418 *HISMX*	*nmt1Δ/NMT1*	Ref^74^

## Data Availability

The docked structures, MD simulation trajectories data are available as free download at 10.5281/zenodo.10477925. Protein expression plasmids of *Pv*NMT and *Hs*NMT1 and *Hs*NMT2 are available at https://www.ssgcid.org/available-materials/ssgcid-proteins/ through material transfer agreement (MTA). All additional data are available from the corresponding authors on reasonable request.
